# Could atmospheric temperature impact on adequate colon cleansing for colonoscopy? An observational, single-institution study

**DOI:** 10.1007/s00384-023-04393-x

**Published:** 2023-04-13

**Authors:** Francesco Maione, Michele Manigrasso, Marco Milone, Nicola Gennarelli, Rosa Maione, Grazia Cantore, Alessia Chini, Giovanni Domenico De Palma

**Affiliations:** 1 Department of Clinical Medicine and Surgery, “Federico II” University of Naples, Via Pansini 5, 80131 Naples, Italy; 2 Department of Advanced Biomedical Sciences, “Federico II” University of Naples, Via Pansini 5, 80131 Naples, Italy

**Keywords:** Colonoscopy, Colon cleansing, Atmospheric temperature, Risk factors

## Abstract

**Purpose:**

Several risk factors affecting the adequacy of colon cleansing have been proposed during the last decades. However, less is known about the impact that atmospheric aspects could have on adequacy of the bowel cleansing. The study aimed to investigate if the atmospheric temperature could impact on the bowel cleansing during colonoscopy.

**Methods:**

A prospective maintained database of the colonoscopies performed since 1^st^ August 2017 to 31^st^ March 2020 was retrospective reviewed. The primary outcome of the study was to identify if the atmospheric temperature was associated with inadequate colon cleansing during colonoscopy. Secondary outcome was to identify the other factors associated with an inadequate colon cleansing.

**Results:**

One thousand two hundred twenty patients were enrolled. High atmospheric temperature (> 25 °C) significantly influenced the colon cleansing (p < 0.0001). Adequate colon cleansing was negatively influenced by gender (female patients were associated with higher colon cleansing rate, p = 0.013), diabetes (p < 0.0001), previous pelvic surgery (p = 0.001), use of Beta-Blocker (p = 0.001), anti-platelet (p = 0.017), angiotensin converting enzyme inhibitors (p = 0.001), the adoption of 4 L Poly Ethylene Glycol solution (p = 0.009), single-dose regimen (p < 0.0001) low patients’ compliance (p < 0.0001), higher age and body mass index (p < 0.0001 and p = 0.025), lower education levels (p < 0.0001). On the contrary, admission to the ward to perform bowel preparation positively impacted on colon cleansing (p = 0.002).

**Conclusion:**

Atmospheric temperature could play an important role in the colon cleansing during colonoscopy, being high temperature (> 25 °C) associated with lower rate of adequate bowel cleansing. However, being this relationship never studied before, these results must be confirmed by other studies.

## Introduction

Adequate colon cleansing is the most important factor influencing the quality of a colonoscopy [1, 2].

Several risk factors affecting the adequacy of colon cleansing have been proposed during the last decades, including diabetes mellitus, neurologic disorders, age, body mass index (BMI), sex, drugs [3–8].

However, less is known about the impact that atmospheric aspects could have on adequacy of the bowel cleansing.

Thus, the aim of the study is to investigate if the atmospheric temperature could impact on the bowel cleansing during colonoscopy.

## Materials and methods

A prospective maintained database of the colonoscopies performed since 1^st^ August 2017 to 31^st^ March 2020 was retrospective reviewed. The study findings have been reported in compliance with the STrengthening the Reporting of OBservational studies in Epidemiology (STROBE) checklist [9].

All the indications to colonoscopies were included in the present study; exclusion criteria were the presence of solid stools in the rectum, the technical inability to complete the colonoscopy or no patient’s tolerance.

Data about age, gender, BMI, presence of diabetes or neurologic disease, previous abdominal or pelvic surgery, the intake of calcium channel blocker or beta-blocker, antiplatelet drugs or anticoagulants, Angiotensin Converting Enzyme (ACE) inhibitors were recorded. Furthermore, education level as a marker of socioeconomic status was registered.

Data about bowel preparation were recorded. Specifically, data about bowel purge were recorded, distinguishing between 4 L (L) of Poly Ethylene Glycol (PEG) solution and 1L of hyperosmolar PEG solution and between single-dose or split-dose regimen. The patient compliance was assessed defining an adequate compliance as the intake of liquid diet and ≥ 75% of the bowel preparation.

All the colonoscopies were performed by expert endoscopists, who had performed at least 1000 colonoscopies.

To analyse colon cleansing, the Boston Bowel Preparation Scale (BBPS) was adopted [10]. Adequate bowel cleansing was defined as a total BBPS > 5, according to the current literature [11].

Finally, data about the month, season and atmospheric temperature were recorded.

Specifically, the atmospheric temperature was assessed by the MeteoApp^®^ for iPhone (Apple Inc, Cupertino, CA, USA).

### Outcomes assessment

Outcomes were divided into primary and secondary outcomes.

The primary outcome of the study was to identify if the atmospheric temperature was associated with inadequate colon cleansing during colonoscopy.

Secondary outcome was to identify the other factors associated with an inadequate colon preparation.

### Statistical analyses

Statistical analyses were performed using SPSS 26.0 (SPSS Inc., Chicago, IL, USA). Continuous variables were expressed as means ± standard deviation (SD); categorical variables were expressed as percentage (%). Continuous variables were compared by the Mann–Whitney U test and t-test and categorical variables are compared by the Chi-square test.

When the minimum expected value was less than five, we adopted the Fisher’s exact test. A *p* value of < 0.05 was defined as statistically significant.

A multivariate logistic regression analysis (stepwise method) was adopted to identify factors independently associated to inadequate right colon cleansing, expressed by Odds Ratio (OR) and 95% Confidence Interval (95% CI).

## Results

Our analysis included 1256 patients. Of them 8 patients were excluded for the impossibility to complete the endoscopy for the presence of an obstructive tumour in the recto-sigmoid junction, 14 for the presence of solid stools in the rectum, 4 for the presence of patient’s intolerable abdominal pain during the procedure. Thus, 1228 patients were enrolled in the final analysis.

Demographic and colonoscopy-related data are shown in Table 1. Of the 1228 included patients, 548 (44.6%) were female, mean age was 58.71 ± 14.26, mean BMI was 26.30 ± 4.44. 148 (12.1%) patients were affected by diabetes, 32 (2.6%) by neurologic disorders. 253 patients (20.6%) underwent previous pelvic surgery and 558 (45.5%) previous abdominal surgery. About drugs, Calcium channel blocker was taken by 98 (8%) patients, beta-blocker by 206 (16.8%), antiplatelets by 236 (19.2%), anticoagulants by 28 (2.3%) and ACE-inhibitors by 329 (26.8%). About education level, 236 patients (19.2%) attended the elementary school, 298 (24.3%) the secondary school, 468 (38.1%) the high school and 226 (18.4%) the university.

**Table 1:** Demographic characteristics of the included patients and bowel preparation data

**Characteristics**	**No of patients (%)**
Patients	1128
Male	580 (55.4)
Female	548 (44.6)
Age (years)	58.71 ± 14.26
BMI (kg/m^2^)	26.30 ± 4.44
Comorbidities	
Diabetes	148 (12.1)
Neurologic disorders	32 (2.6)
Previous pelvic surgery	253 (20.6)
Previous abdominal surgery	558 (45.5)
Drugs use	
Calcium Channel Blocker	98 (8)
Beta- Blocker	206 (16.8)
Antiplatelets	236 (19.2)
Anticoagulants	28 (2.3)
ACE Inhibitors	329 (26.8)
Education level	
Elementary school	236 (19.2)
Secondary school	298 (24.3)
High school	468 (38.1)
University	226 (18.4)
Hospitalization	162 (13.2)
Bowel preparation type	
4L PEG solution	1130 (92)
1L hyperosmolar PEG solution	98 (8)
Timing of bowel preparation	
Single-dose regimen	1030 (83.9)
Split-dose regimen	198 (16.1)

*BMI* Body Mass Index, *ACE* Angiotensin Converting Enzyme, *PEG* Poly Ethylene Glycol

One hundred sixty-two patients (13.2%) were hospitalized the day before colonoscopy to undergo bowel purge; 4L PEG solution was administered to 1130 patients (92%), and single-dose regimen was adopted in 1030 patients (83.9%). Compliance was satisfactory in 1056 (86%) of cases. Adequate colon cleansing was obtained in 990 (80%) cases.

Primary and secondary outcomes are shown in Table 2.

**Table 2:** Univariate analysis on covariates influencing the adequate right colon cleansing

**Covariates**	**P value**
Atmospheric temperature	** < 0.0001**
Gender	**0.013**
Age (years)	** < 0.0001**
BMI (kg/m^2^)	**0.025**
Comorbidities	
Diabetes	** < 0.0001**
Neurologic disorders	0.073
Previous pelvic surgery	**0.001**
Previous abdominal surgery	0.053
Drugs use	
Calcium Channel Blocker	0.437
Beta- Blocker	**0.001**
Antiplatelets	**0.017**
Anticoagulants	0.342
ACE Inhibitors	**0.001**
Education level	** < 0.0001**
Hospitalization	**0.002**
Bowel preparation type	**0.009**
Timing of bowel preparation	** < 0.0001**

*BMI* Body Mass Index, *ACE* Angiotensin Converting Enzyme

Atmospheric temperature significantly influenced the colon cleansing (p < 0.0001). Dividing the patients into 6 clusters according to the atmospheric temperature, results showed a higher rate of patients with an inadequate colon cleansing when the temperature was high (> 25 °C).

About season, results confirmed that during the summer the rate of bowel cleansing was low if compared with the other seasons (72.3%, p < 0.0001) and especially during July (63.2%, p < 0.0001).

Adequate colon cleansing was negatively influenced by gender (female patients were associated with higher colon cleansing rate, p = 0.013), diabetes (p < 0.0001), previous pelvic surgery (p = 0.001), use of Beta-Blocker (p = 0.001), anti-platelet (p = 0.017), ACE-inhibitors (p = 0.001), the adoption of 4L PEG solution (p = 0.009), single-dose regimen (p < 0.0001) and low patients’ compliance (p < 0.0001). Similarly, higher age and BMI negatively impacted on the bowel preparation (p < 0.0001 and p = 0.025, respectively). Regarding education level, lower education levels were associated with a higher rate of inadequate colon cleansing in a significant way (p < 0.0001). On the contrary, admission to the ward to perform bowel preparation positively impacted on colon cleansing (p = 0.002).

Interestingly, the presence of neurologic disorders (p = 0.073), abdominal surgery (p = 0.053), the use of anti-coagulants drugs (p = 0.342) and Calcium Channel Blocker (p = 0.437) did not significantly impact on the adequate colon cleansing.

Multivariate analysis showed that the presence of diabetes (OR 2.341; 95% CI 1.531–3.578; p < 0.0001), the admission to the ward to perform bowel preparation (OR 1.633; 95% CI 1.088–2.450; p = 0.018), single-dose regimen of the bowel preparation (OR 0.382; 95% CI 0.224–0.652; p < 0.0001), low education level (OR 0.798; 95% CI 0.678–0.941; p < 0.0001), low compliance to the bowel preparation (OR 0.372; 95% CI 0.253–0.545; p < 0.0001) and the month of the year (OR 0.946; 95% CI 0.905–0.988; p = 0.013) represented independent factors related to inadequate colon cleansing.

Results of multivariate analysis are shown in Table 3.

**Table 3:** Multivariate analysis on covariates influencing the adequate right colon cleansing

**Covariates**	**P value**	**OR (95% CI Intervals)**
Month	0.013	0.946 (0.905–0.988)
Diabetes	0.004	2.341 (1.531–3.578)
Admission to the ward	0.018	1.633 (1.088–2.450)
Single-dose regimen	< 0.0001	0.382 (0.224–0.652)
Education level	< 0.0001	0.798 (0.678–0.941)
Compliance to the bowel prep	< 0.0001	0.372 (0.253–0.545)

## Discussion

Inadequate bowel cleansing plays a critical role in the efficacy and safety of the colonoscopy: in fact, a good bowel preparation allows the detection of benign and malignant colonic lesions and optimizes cecal intubation while a poor bowel preparation is associated with prolonged procedure time, difficult progression, failure to detect colorectal lesions and increased risk of complication [1, 2].

Several risk factors for the inadequate colon cleansing have been proposed in the current literature.

Patients related factors included increasing age [12, 13], obesity [8, 14, 15], male gender [7, 16], the presence of comorbidities [13, 17–19], abdominal or pelvic surgery, health literacy [20–23] and some medications (opioids, Calcium channel blocker and antidepressants) [6–8].

It is well known that all these patients’ related factors associated with inadequate colon cleansing were strictly connected with an impaired gastrointestinal transit.

Interestingly, male gender has been described as an independent predictor of inadequate colon cleansing [7, 16, 20]. The reason of this aspect could lay in the less health-consciousness compared with the women. This difference could result in a lower adherence to the bowel preparation protocols.

Regarding the association between bowel preparation and BMI, Laurie et al. [24] in their study reported that obese patients, which have an increased rate of colorectal lesions and an increased risk of complications from colonoscopy, are more likely to have poor bowel preparation at colonoscopy compared to participants with a healthy weight regardless the type of oral bowel preparation.

Similarly, low education level, as a marker of low socioeconomic status, could be related with a low comprehension of the dietary restriction and bowel preparation prior to the colonoscopy, with a consequent low adherence to the preparation protocols.

Our results confirmed these data. In fact, we are able to confirm that male gender significantly affected the adequate bowel cleansing, as well as the presence of diabetes, previous pelvic surgery, the increasing age, BMI, and education status. On the contrary, the presence of neurologic disorders, abdominal surgery, the use of anti-coagulants drugs and Calcium Channel Blocker did not significantly impact on the adequate colon cleansing.

About these factors, we have to consider that these factors are directly related to the patients’ condition, and for this reason, they are not modifiable.

Regarding procedure related risk factors, several studies have proposed that low-volume hyperosmolar PEG and split-dose regimen were associated with an higher rate of adequate bowel cleansing [25–31].

In a prospective, multicentre, observational study, Maida et al. [25] compared 1L preparation to 4L and 2L- PEG solutions in a real-life setting. By pooling together 1289 patients, the Authors concluded that 1L solution was effective and well tolerated, also showing it was an independent predictor of overall cleansing success, high-quality cleansing of the right colon and of tolerability.

Similar results were obtained by Frazzoni et al. [26] in a propensity score matching analysis on 1004 patients. The Authors found a higher rate of adequate colon cleansing in the 1L-PEG solution group.

In a randomized pilot study, Hernandez et al. [27] compared the efficacy of bowel preparation volume size in hospitalized patients, randomly assigning 25 patients to receive a high, medium or low-volume preparation. Results showed that low-volume preparation should be preferred in hospitalized patients, because of its tolerability and comparable bowel cleanliness when compared to higher volume preparation.

Comparing whole versus split-dose regimen, El Sayed et al. [28], in a randomized trial on 187 patients demonstrated that split-dose regimen provided better quality colon cleansing and higher compliance than whole dose regimen.

More recently, Mohamed et al. [29] randomized 287 patients to receive a single 4L PEG or split-dose PEG preparation. The results supported the use of a split-dose regimen for superior bowel cleanliness.

Similarly, Shan et al. [30], in a randomized controlled trial on 295 patients, demonstrated that for morning colonoscopy, split-dose 2L PEG was superior to single-dose regimen in bowel preparation and patients tolerability and satisfaction.

A recent systematic review and meta-analysis on 17 randomized clinical trials [31], showed that low-volume split-dose regimen appeared to be as effective as high-dose, but better tolerated, with superior compliance.

As above described, the importance of adoption of low-volume, split-dose preparation was related with the patients satisfactory and compliance. The latter, regardless of the type of purgative used, represented alone an important determinant of quality in bowel preparation [7, 21].

In our study, the majority of patients underwent 4L PEG solution (1130, 92%), and single-dose regimen (1030, 83.9%). Nevertheless, the compliance was high (1056, 86%).

In this setting, our results showed that the adoption of 4L PEG solution and single-dose regimen were associated with a higher rate of inadequate colon cleansing (p = 0.009 and p < 0.0001, respectively), confirming the results of the current literature.

Although these risk factors for inadequate bowel preparation have been well investigated, no Author has investigated the atmospheric temperature as a risk factor.

In fact, to our best knowledge this is the first study in which the atmospheric temperature has been investigated as a risk factor for inadequate colon cleansing. Regarding this aspect, our results demonstrated that the atmospheric temperature significantly influenced the colon cleansing (p < 0.0001). Specifically, our study demonstrated that the lowest rate of adequate bowel cleansing was present when then external temperature was above 25 °C (68.5%). Further analyses demonstrated that July represented the month in which the patients had the worst bowel cleansing. The rationale of these results could lay in the dehydration related to the high temperature during the summer. In fact, in case of warm and humid climate, the body temperature raises and the physiological response to this is sweating, which leads to cooling through evaporation. The maintenance of high sweat rate causes progressive dehydration [32].

Major limitation has to be addressed to our study. In fact, this is a retrospective review of a single-institution database. Thus, the study could be affected by inherent selection bias.

## Conclusion

Although the retrospective design of this study, we can conclude that atmospheric temperature could play an important role in the colon cleansing during colonoscopy, being high temperature (> 25 °C) associated with lower rate of adequate bowel cleansing. However, being this relationship never studied before, these results must be confirmed by other studies. If confirmed, these results could lead to consider the atmospheric temperature as a risk factor for inadequate colon cleaning and to consider patients as high-risk. This could lead to further dietary modification before the colonoscopy and to modify the bowel preparation protocols.

## Data Availability

Data are available to the corresponding Author.
